# Hyposalinity reduces coordination and adhesion of sea urchin tube feet

**DOI:** 10.1242/jeb.245750

**Published:** 2023-06-30

**Authors:** Andrew J. Moura, Austin M. Garner, Carla A. Narvaez, Jack P. Cucchiara, Alyssa Y. Stark, Michael P. Russell

**Affiliations:** ^1^ Department of Biology, Villanova University, Villanova, PA 19085, USA; ^2^ Department of Biology and BioInspired Syracuse, Syracuse University, Syracuse, NY 13244, USA; ^3^ Friday Harbor Laboratories, University of Washington, Friday Harbor, WA 98250, USA; ^4^ Department of Biology, Rhode Island College, Providence, RI 02908, USA

**Keywords:** Climate change, Extreme climatic events, Echinoderms, Muscular hydrostats, Precipitation

## Abstract

Climate change will increase the frequency and intensity of low-salinity (hyposalinity) events in coastal marine habitats. Sea urchins are dominant herbivores in these habitats and are generally intolerant of salinity fluctuations. Their adhesive tube feet are essential for survival, effecting secure attachment and locomotion in high wave energy habitats, yet little is known about how hyposalinity impacts their function. We exposed green sea urchins (*Strongylocentrotus droebachiensis*) to salinities ranging from ambient (32‰) to severe (14‰) and assessed tube feet coordination (righting response, locomotion) and adhesion [disc tenacity (force per unit area)]. Righting response, locomotion and disc tenacity decreased in response to hyposalinity. Severe reductions in coordinated tube foot activities occurred at higher salinities than those that affected adhesion. The results of this study suggest moderate hyposalinities (24–28‰) have little effect on *S. droebachiensis* dislodgement risk and survival post-dislodgment, while severe hyposalinity (below 24‰) likely reduces movement and prevents recovery from dislodgment.

## Teaser

**Summary:** We demonstrate that sea urchin righting response, locomotion and adhesion are negatively impacted by hyposalinity, an environmental challenge expected to increase in intensity and frequency under global climate change.

## INTRODUCTION

Climate change will increase the frequency and intensity of extreme climatic events (ECEs) such as heatwaves, droughts, storms and intense precipitation (Coumou and Rahmstorf, 2012; Smith, 2011). Organisms may be particularly susceptible to their effects because they likely lack adaptations for these unprecedented events (Holt, 2008). In marine ecosystems, the biological consequences of thermal stress associated with heatwaves have received considerable attention (Smith et al., 2023), yet the effects of osmotic stress associated with low salinity (hyposalinity) following storm-related freshwater input remain poorly resolved (Cheng et al., 2016). Drastic changes in salinity are particularly problematic for stenohaline organisms, such as sea urchins, that possess little to no capacity to osmoregulate (Burnett et al., 2002; Russell, 2013; Vidolin et al., 2007).

Sea urchins are dominant herbivores and ecosystem engineers in many benthic communities (Davidson and Grupe, 2015; Steneck, 2020). In temperate ecosystems, sea urchins are capable of reorganizing community structure by overgrazing macroalgae, decreasing primary productivity and habitat for many economically important species (Steneck, 2020). Understanding how environmental stressors induced by ECEs, like periods of hyposalinity, will affect sea urchin populations is critical to predict the impact of climate change on the ecosystem services provided by temperate rocky reef systems.

Sea urchin survival and their success as herbivores are largely mediated by their tube feet, muscular hydrostatic extensions of the water vascular system involved in attachment, locomotion, feeding, respiration and sensing (Flammang, 1996; Leddy and Johnson, 2000; Lesser et al., 2011; Rodriguez, 2003). Tube feet generally consist of an extensible stalk terminating in a disc containing a duo-gland adhesive system that secretes an adhesive and de-adhesive to attach and detach to substrata, respectively (Flammang, 1996).

The green sea urchin (*Strongylocentrotus droebachiensis*) is one of few euryhaline sea urchin species (Russell, 2013), occurring in the Northern Atlantic, Northern Pacific and Arctic Oceans. It is a dominant grazer and an important fisheries species (Scheibling et al., 2020). Found in intertidal, subtidal and estuarine habitats throughout its range, *S. droebachiensis* is capable of acclimating to severe hyposalinity (Drouin et al., 1985; Himmelman et al., 1984; Russell, 2013; Scheibling et al., 2020), but qualitative observations of tube feet under these conditions suggest impaired function (Russell, 2013).

Here, we assessed the impacts of hyposalinity on *S. droebachiensis* tube foot function. We quantified individual [disc tenacity (adhesive force per unit area)] and coordinated tube feet function [righting response (ability to right after inversion)] and locomotion] along a salinity gradient. We hypothesized that both parameters would decline similarly in response to hyposalinity.

## MATERIALS AND METHODS

### Sea urchin collection and experimental design

We collected green sea urchins, *Strongylocentrotus droebachiensis* (Müller 1776) (44.95±8.46 mm diameter, *n*=30), from a depth of 9 m near Friday Harbor Laboratories on San Juan Island, Washington (48°32′26.2392″N, 123°0′40.3128″W) on 31 October 2022. Sea urchins were brought to the laboratory and divided into three size classes based on diameter (30–40, 40–50 and 50–60 mm). Sea urchins from each size class were equally divided into 10 containers (18.4×13.3×13.3 cm, *n*=3 per container). Containers were filled with ∼3 l of filtered natural seawater that was oxygenated with air diffusers and placed in a flow-through sea table at ambient temperature (11.3±0.3°C).

Each container was randomly assigned to an even integer treatment salinity ranging from 14 to 32 parts per thousand (‰). Containers were then lowered to their treatment salinity at a rate of 0.98±0.17‰ 10 min^−1^ by adding chilled reverse osmosis (RO) water. Final treatment salinity was ±0.5‰ of the target salinity. Tube foot function was assessed after sea urchins were kept in their target treatment salinity for 24 h (Russell, 2013). To remove accumulated solid and nitrogenous waste, partial seawater changes were conducted after 6 and 12 h with seawater matching the treatment salinity for each container. Sea urchins were not fed during the experiment.

### Tube foot function

Righting response, locomotion and disc adhesive force were assessed for each sea urchin in seawater of their respective treatment salinity. Seawater of each treatment salinity was made by diluting filtered natural seawater with RO water. Disc area, used to calculate disc tenacity, was measured before the start of the experiment in filtered natural seawater at ambient salinity.

### Righting response

Righting response is the time required for a sea urchin to completely flip from their inverted aboral body axis to their oral body axis (i.e. mouth facing the substrate). It is a common metric of neuromuscular coordination in sea urchins, requiring coordination of tube feet and spines (Collin et al., 2018). Sea urchins were placed, inverted, in a 10 gallon (∼38 l) glass aquarium filled with ∼8 l of seawater and the time (s) required for sea urchins to right themselves was recorded. Many sea urchins failed to right in 300 s, suggesting they could not right themselves within a biologically relevant time frame. In these instances, we recorded righting time as 300 s. We converted the righting response (righting time, s) to an activity coefficient (AC, s^−1^) using the formula:
(1)

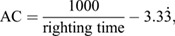
such that faster righting times correspond to higher AC, slower righting times correspond to lower AC, and failure to right in 300 s corresponds to an AC of 0 (Barker and Russell, 2008).

### Locomotion

Locomotor performance was assessed by placing sea urchins at one end of a 10 gallon (∼38 l) glass aquarium and dispensing 0.5 ml of 2% bleach in seawater at the ambulacral column opposite to the desired direction of movement. Preliminary trials indicated sea urchins actively move away from a dilute bleach solution. We recorded sea urchin movement with a DSLR camera (Nikon D5600, Nikon USA, Melville, NY USA) positioned beneath the aquarium with a 1 cm^2^ scale. One frame per second for the first 30 s of movement was isolated (resulting in 30 frames) and analyzed in ImageJ using the MTrackJ plugin (Meijering et al., 2012). We tracked the center of the sea urchin's exposed jaw across the 30 frames and calculated instantaneous speed using the distance covered and time taken from one frame to the next. Maximum locomotor speed (cm s^−1^) was calculated as the maximum instantaneous frame-to-frame speed recorded in each trial.

### Disc tenacity

Disc tenacity (maximum adhesive force per unit area, MPa) of oral tube feet was assessed using the method described by Narvaez et al. (2022). Average oral tube foot disc area was measured by restraining sea urchins in a sponge-packed PVC collar topped with a glass Petri dish held in a 3 l container of ambient (32‰) seawater. Once at least 10 tube feet discs had attached to the glass, a photograph with a 1 mm scale was taken (Tough TG-6, Olympus Corporation of the Americas, Center Valley, PA, USA). The disc area of 10 randomly selected attached discs was calculated using ImageJ and used to estimate the mean disc area for each individual (Abramoff et al., 2004; Stark et al., 2020).

Disc adhesive force was measured by placing a sea urchin in a sponge-packed PVC collar mounted on the side of a 3 l container of seawater. A glass capillary tube (1.5 mm diameter) attached to a handheld digital force gauge (FGV-5XY, Shimpo Instruments, Glendale Heights, IL, USA) by a monofilament thread was presented to the oral tube feet. Once a single tube foot disc had attached to the side of the capillary tube, adhesive force was measured by pulling the force gauge at a constant rate by hand until the disc detached from the capillary tube (Fig. S1). The same researcher (A.J.M.) conducted all adhesion trials (Narvaez et al., 2022). Adhesive force was measured for three different tube feet per sea urchin. If no tube feet attached within 5 min, the trial was discontinued, and the sea urchin was omitted from data analysis. For each sea urchin, the highest value of each of three trials was used to calculate disc tenacity by dividing the maximum adhesive force by the mean disc area of that sea urchin.

### Statistical analysis

All analyses were performed in R (https://www.r-project.org/). We used general additive models (GAMs – package *mgcv*; Wood, 2011) to compare the relationship between salinity and tube foot functionality among the three performance metrics (activity coefficient, maximum speed, disc tenacity). Salinity was modeled as a thin plate smoothing spline and smoothing parameters selected using generalized cross-validation criteria. The degree of non-linearity of the models is provided as effective degrees of freedom (e.d.f.), whereby higher e.d.f. corresponds to a more non-linear relationship. A linear regression showed no significant relationship between sea urchin diameter and any of the tube foot functionality metrics (activity coefficient: *t*_28_=−0.746, *P*=0.462; maximum locomotor speed: *t*_28_=0.466, *P*=0.645; disc tenacity: *t*_27_=−0.115, *P*=0.878); thus, salinity was included as the only explanatory variable. Assumptions of normality and homoscedasticity were assessed graphically (Boldina and Beninger, 2016). Normal distribution of residuals was assessed with a histogram and quantile–quantile plot. Homogeneity of variances was verified by plotting the model residuals versus the fitted values (Fig. S2). Activity coefficient data violated assumptions; natural log transformation improved assumptions, but the results were qualitatively similar to those with non-transformed data (Figs S2 and S3). We report the latter for ease of interpretation and visualization.

The mean value of each performance metric was calculated for each salinity and the greatest mean was used as maximum performance. Performance of each sea urchin was then graphed as a percentage of maximum performance to facilitate comparisons between performance metrics.

## RESULTS AND DISCUSSION

**Fig. 1.**
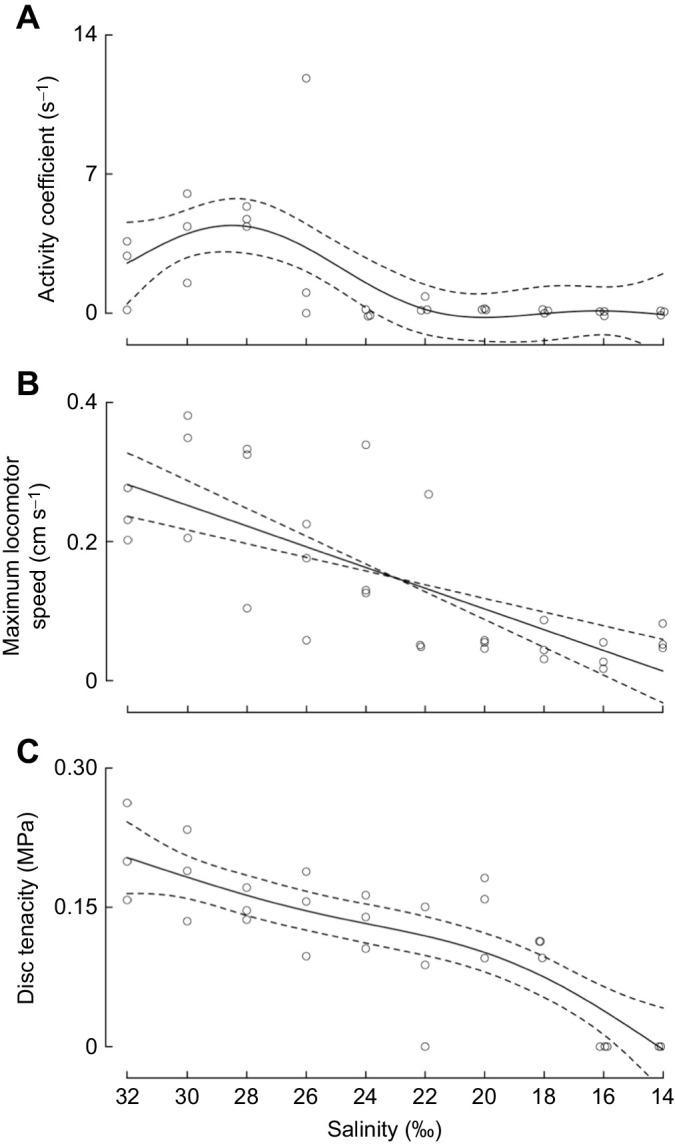
**Sea urchin tube foot adhesion and coordination with decreasing salinity.** Estimated smooths and scatter plots for (A) activity coefficient, (B) maximum locomotor speed and (C) disc tenacity of sea urchins kept in salinities from 32‰ (ambient) to 14‰ seawater for 24 h. The solid black lines represent the estimate of the smooth and dashed lines represent two standard errors above and below the smooth.

**Fig. 2.**
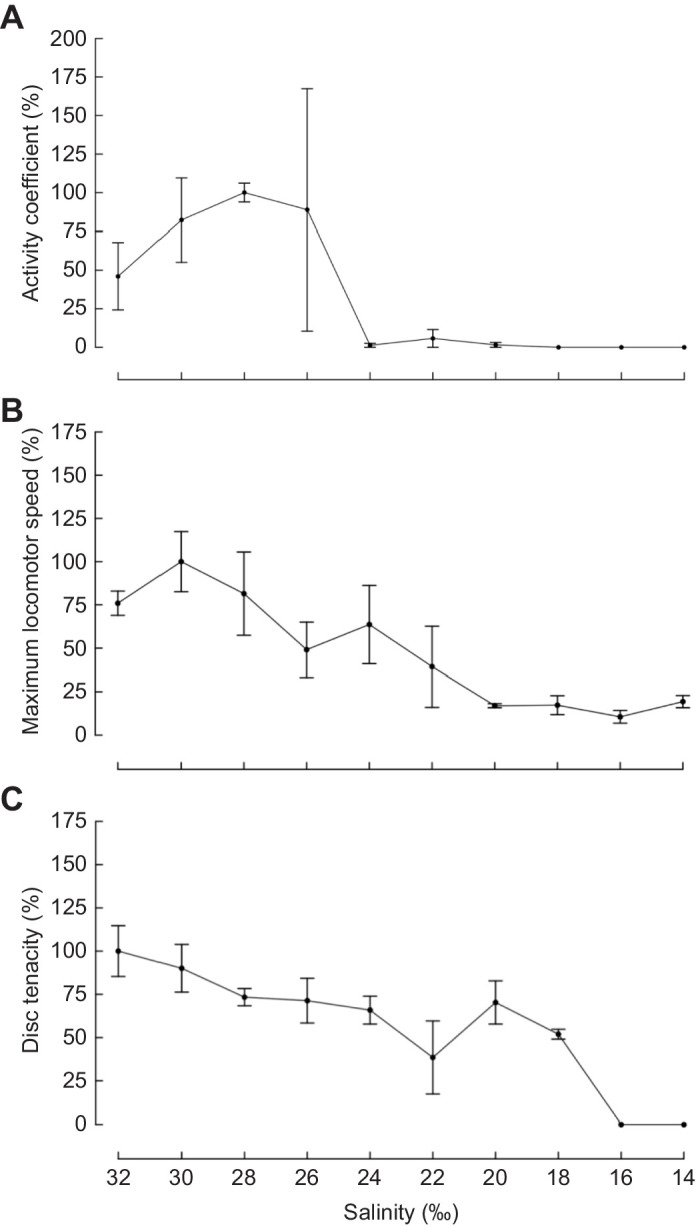
**Performance with decreasing salinity.** Mean percentage of maximum performance for (A) activity coefficient, (B) maximum locomotor speed and (C) disc tenacity for sea urchins kept in salinities from 32‰ (ambient) to 14‰ seawater for 24 h. Data are means±1 s.e.m.

Our results show that sea urchin tube foot adhesion and coordination decline with decreasing salinity in distinct linear and non-linear relationships. The activity coefficient had the least linear relationship of the three metrics (e.d.f.=3.522), resembling a cubic or quartic function (Fig. 1A, Table 1). Maximum locomotor speed had the most linear relationship, with an e.d.f. of 1, suggesting a positive linear relationship between salinity and maximum locomotor speed (Fig. 1B, Table 1). Disc tenacity had an e.d.f. of 2.516, resembling a quadratic or cubic function (Fig. 1C, Table 1). One sea urchin did not extend tube feet during adhesive force measurements at 14‰ and was omitted from data analysis. Furthermore, sea urchins experienced severe reductions in each performance metric at different salinities. Relative to maximum performance, activity coefficient was severely reduced at ≤24‰ (Fig. 2A), locomotion was severely reduced at ≤20‰ (Fig. 2B), and disc tenacity was severely reduced at ≤16‰ (Fig. 2C).

**Table 1.**
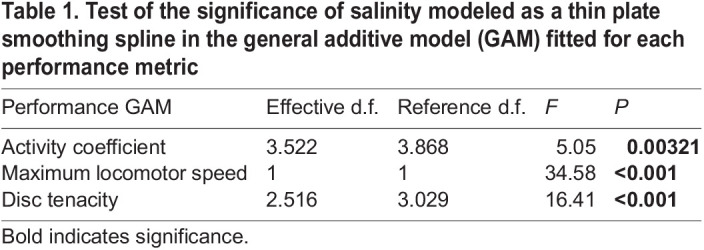
Test of the significance of salinity modeled as a thin plate smoothing spline in the general additive model (GAM) fitted for each performance metric

Reduced tube foot functionality under hyposalinity may be the result of the dilution of ions in seawater important for tube feet neuromuscular activity and adhesion. Calcium, sodium and potassium, for example, are essential for many physiological and cellular activities, including neuromuscular function (Carafoli, 2005; Hill et al., 2012). At lower salinities, these ions may be below the concentrations required for normal neuromuscular function and thus responsible for reduced tube foot coordination. A decrease in the concentration of ions important for neuromuscular function may therefore explain the decline of highly coordinated activities (locomotion, righting response) at higher salinities than disc tenacity. However, calcium is hypothesized to induce secretion of the adhesive in tube feet, and many marine adhesives use non-covalent interactions such as calcium and magnesium bridging to achieve adhesion to the substratum (Hennebert et al., 2015; Lebesgue et al., 2016). Thus, changes in various ion concentrations from hyposalinity may impact the formation, release and composition of the adhesive. Future work is needed to better understand the physiological and cellular mechanisms responsible for reduced tube foot functionality in hyposaline conditions.

The inability to regain attachment and proper orientation after dislodgement from hydrodynamic forces and predators can be fatal to sea urchins (Dayton, 1973, 1975). The observed decline in activity coefficient suggests hyposalinity will aggravate the negative impacts of dislodgment for sea urchins experiencing severe hyposalinity (below 24‰) by reducing the ability to recover proper orientation. The ability to evade high hydrodynamic forces and predators may also decrease under hyposalinity as sea urchins become slower in response to a negative stimulus (Fig. 1B). The effect of severe hyposalinity on sea urchin survival may be partially alleviated by relatively little change in disc tenacity between 18‰ and 28‰ (Figs 1C and 2C). Individual tube feet attached readily to glass capillary tubes in 18‰ seawater and thus may retain sufficient functionality at low salinities to allow sea urchins to remain attached and compensate for reduced adhesion by recruiting more tube feet to the substratum, preventing dislodgment (Narvaez et al., 2022).

Hyposalinity may have a pronounced effect on tube foot functionality that persists after returning to average conditions; thus, future experiments should investigate the potential for recovery of tube foot functionality once sea urchins are returned to average salinity conditions. Sea urchins likely experience multiple hyposalinity events within the same year. Repeated exposures may have a detrimental effect on performance or may result in acclimation where performance increases over time. Indeed, *S. droebachiensis* growth rate acclimates to hyposalinity and populations exposed to hyposalinity exhibit a greater ability to right themselves under hyposalinity than unexposed populations, suggesting local acclimation (Drouin et al., 1985; Himmelman et al., 1984; Russell, 2013). However, tube feet exhibit considerable phenotypic plasticity in performance and morphology (Cohen-Rengifo et al., 2017; Narvaez et al., 2020, 2022; Santos and Flammang, 2007; Stark et al., 2020; Toubarro et al., 2016). Given that plastic traits are often energetically expensive (Dewitt and Scheiner, 2004), maintenance of homeostasis and tube foot performance may present competing energetic demands, resulting in lower tube foot performance under stressors such as hyposalinity. The sea urchins used in this study were collected from a depth of 9 m and although they were likely protected from hyposalinity, we lack long-term salinity measurements along a bathymetric gradient at our collection sites. Therefore, future studies should measure salinity levels within different sea urchin microhabitats and test the capacity for and extent to which sea urchins acclimate to hyposalinity within and between populations with differential exposure to hyposalinity.

Future ECEs associated with global climate change will likely expose sea urchins to more intense and frequent variations in salinity (Nyhagen et al., 2018). Our results suggest moderate hyposalinity (24–28‰) will have limited effects on *S. droebachiensis* performance. Severe hyposalinity (below 24‰) will likely increase dislodgment risk, reduce movement and prevent recovery from dislodgment. However, *S. droebachiensis* is one of few euryhaline sea urchin species and likely represents a ‘best case scenario’ for sea urchin performance under hyposalinity when compared with stenohaline species. Therefore, understanding the performance of stenohaline sea urchin species under hyposalinity and the capacity for performance to acclimate to hyposalinity will strengthen predictions about the consequences of ECEs on these critical members of marine communities.

## Supplementary Material

10.1242/jexbio.245750_sup1 Supplementary information
