# Supplementary material for: Hyposalinity reduces coordination and adhesion of sea urchin tube feet
Source: J Exp Biol. 2023 Jun 30;226(13):jeb245750. doi: 10.1242/jeb.245750 (PMC10323246; doi:10.1242/jeb.245750)
Supplement: Supplementary information [file jexbio-226-245750-s1.pdf]

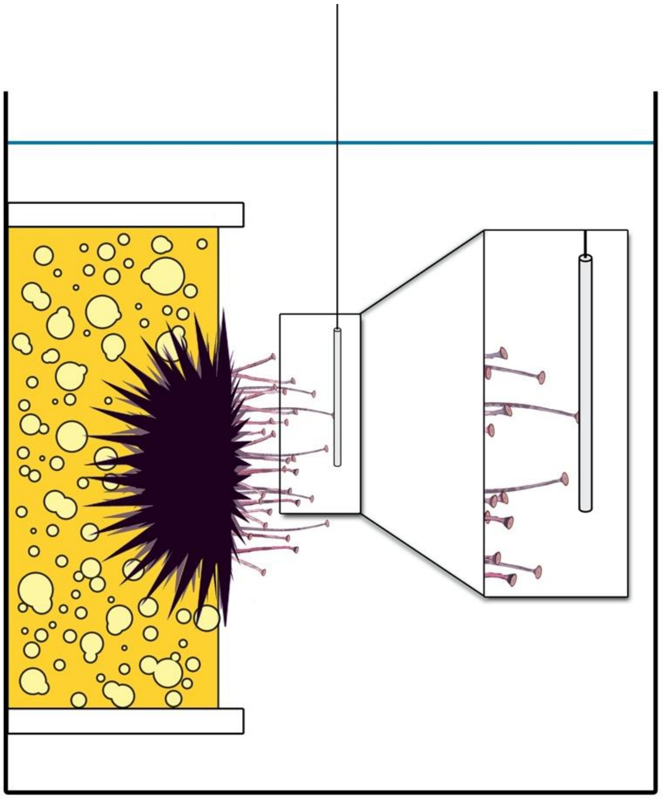

Fig. S1. Adapted from Narvaez et al. (2022). Diagram of the methodology used to measure tube foot disc adhesive force. Sea urchins were restrained by a sponge (yellow), exposing only oral tube feet. The sea urchin inside the sponge was placed within a PVC pipe (white bars holding the yellow sponge) submerged in seawater. The maximum adhesive force of the disc was assessed by a capillary tube attached to a handheld digital force gauge by a monofilament thread. Once a single tube foot was attached, a constant vertical force was applied, and the force required to detach the tube foot was recorded.

Journal of Experimental Biology • Supplementary information

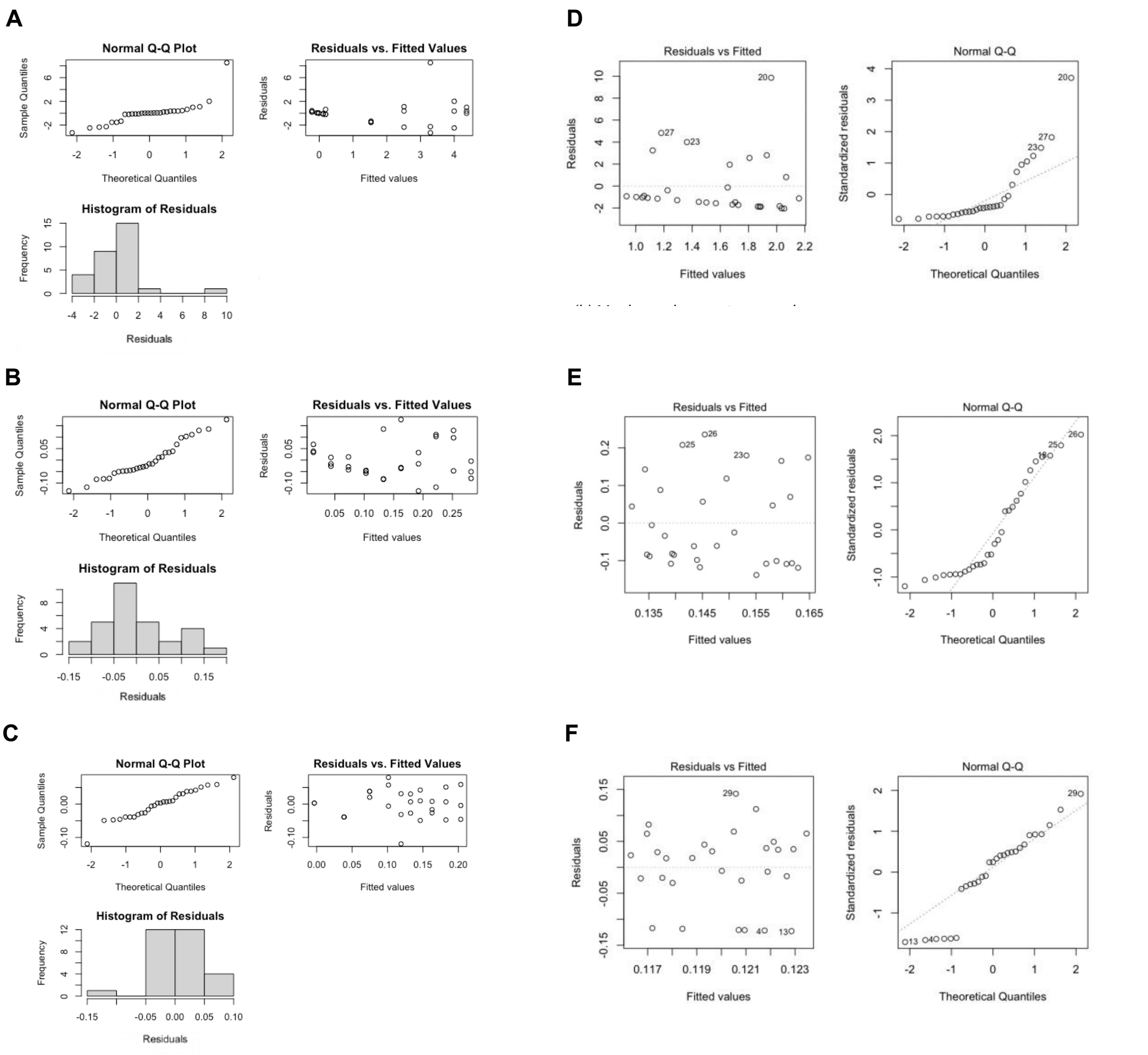

Fig. S2. Assumptions validation of the linear regression of (A) activity coefficient, (B) maximum locomotor speed, and (C) disc tenacity as a function of diameter for sea urchins kept in salinities from 32 (ambient) to 14‰ seawater for 24hr. Assumptions validation of the GAMs for the relationship between salinity (D) activity coefficient, (E) maximum locomotor speed, and (F) disc tenacity of sea urchins kept in salinities from 32 (ambient) to 14‰ seawater for 24hr. Normality assumption shown as a histogram and QQplot of the model residuals. Homoscedasticity shown as a scatterplot of residuals versus fitted values.

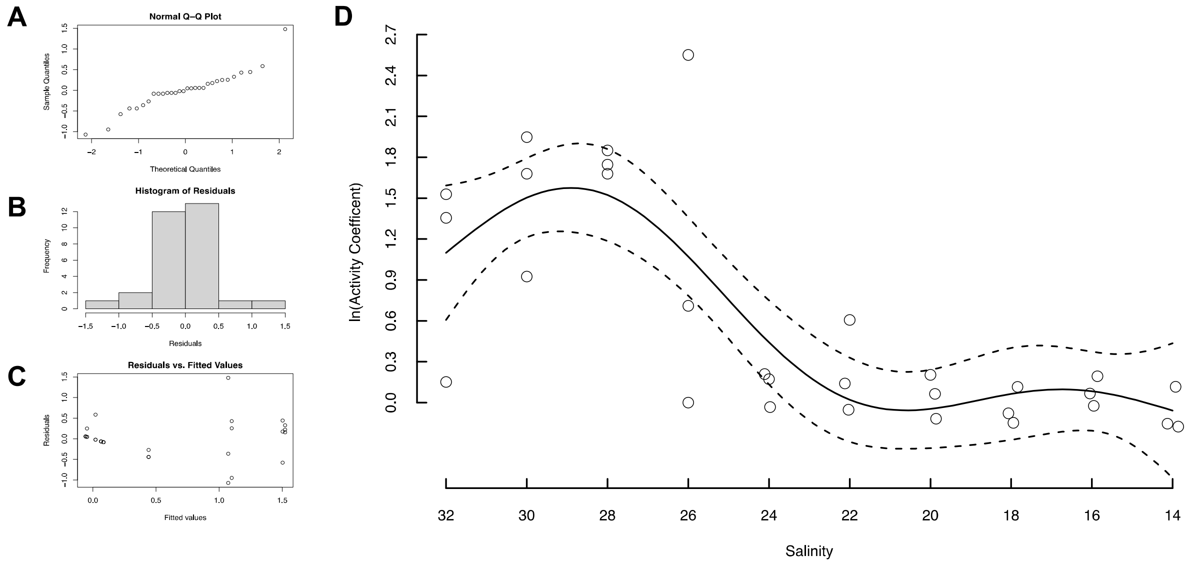

Fig. S3. Assumptions validation (A, B, C) and plot (D) of the GAM for the relationship between salinity and natural log transformed activity coefficient data for sea urchins kept in salinities from 32 (ambient) to 14‰ seawater for 24hrs. Normality assumption was assessed with a QQplot (A) and histogram (B) of the model residuals. Homoscedasticity was assessed with a scatterplot of residuals versus fitted values (C). The estimated smooth and scatter plot (D) for natural log transformed activity coefficient is represented by a solid black line and dashed lines represent two standard errors above and below the smooth.

Journal of Experimental Biology • Supplementary information
